# Ecological Sexual Dimorphism and Environmental Variability within a Community of Antarctic Penguins (Genus *Pygoscelis*)

**DOI:** 10.1371/journal.pone.0090081

**Published:** 2014-03-05

**Authors:** Kristen B. Gorman, Tony D. Williams, William R. Fraser

**Affiliations:** 1 Department of Biological Sciences, Simon Fraser University, Burnaby, British Columbia, Canada; 2 Polar Oceans Research Group, Sheridan, Montana, United States of America; Phillip Island Nature Parks, Australia

## Abstract

**Background:**

Sexual segregation in vertebrate foraging niche is often associated with sexual size dimorphism (SSD), i.e., *ecological sexual dimorphism*. Although foraging behavior of male and female seabirds can vary markedly, differences in isotopic (carbon, δ^13^C and nitrogen, δ^15^N) foraging niche are generally more pronounced within sexually dimorphic species and during phases when competition for food is greater. We examined ecological sexual dimorphism among sympatric nesting *Pygoscelis* penguins asking whether environmental variability is associated with differences in male and female pre-breeding foraging niche. We predicted that all *Pygoscelis* species would forage sex-specifically, and that higher quality winter habitat, i.e., higher or lower sea ice coverage for a given species, would be associated with a more similar foraging niche among the sexes.

**Results:**

P2/P8 primers reliably amplified DNA of all species. On average, male *Pygoscelis* penguins are structurally larger than female conspecifics. However, chinstrap penguins were more sexually dimorphic in culmen and flipper features than Adélie and gentoo penguins. Adélies and gentoos were more sexually dimorphic in body mass than chinstraps. Only male and female chinstraps and gentoos occupied separate δ^15^N foraging niches. Strong year effects in δ^15^N signatures were documented for all three species, however, only for Adélies, did yearly variation in δ^15^N signatures tightly correlate with winter sea ice conditions. There was no evidence that variation in sex-specific foraging niche interacted with yearly winter habitat quality.

**Conclusion:**

Chinstraps were most sexually size dimorphic followed by gentoos and Adélies. Pre-breeding sex-specific foraging niche was associated with overall SSD indices across species; male chinstrap and gentoo penguins were enriched in δ^15^N relative to females. Our results highlight previously unknown trophic pathways that link *Pygoscelis* penguins with variation in Southern Ocean sea ice suggesting that each sex within a species should respond similarly in pre-breeding trophic foraging to changes in future winter habitat.

## Introduction

Intra-population variation in ecological niche, sensu [1], is widespread in nature [2], [3] however, the ecological and evolutionary causes and consequences of such individual variability remain poorly understood [4]. Sexual segregation in foraging niche of vertebrates is a relatively well-studied example of intra-population ecological differences [5] that is often considered to be mediated by sexual size dimorphism (SSD) [6], [7]. In theory, the evolution and maintenance of SSD reflects sexual variance in the adaptive process in which fitness of adult males and females is maximized at differing body sizes [6], with major functional hypotheses including sexual and/or fecundity selection [8]–[10]. Ecological niche divergence [11], [12] is an alternative hypothesis, generally considered secondary, which evolves often as a consequence of existing SSD [6], [10], i.e., *ecological sexual dimorphism* [4].

Studies of marine birds have documented differences in foraging behavior, i.e., spatial and temporal variation in activity budgets of males and females within SSD-associated systems, e.g., [13], [14]. However, sexually monomorphic species also exhibit sex-specific foraging, e.g., [15], [16], raising questions about the importance of structural size in mediating sexual differences in foraging behavior, see [15] for review. Recent studies have advanced the issue using naturally occurring ratios of carbon (^13^C/^12^C, δ^13^C) and nitrogen (^15^N/^14^N, δ^15^N) stable isotope (SI) signatures to more rigorously assess whether males and females occupy different foraging niches [17]–[19] based on a time-integrated, biogeochemical parameter that specifically reflects assimilated prey [20]–[22]. In a recent review, Phillips et al. [19] concluded that a) isotopic differences between male and female seabirds were extremely rare in sexually monomorphic species, strengthening the idea that sexual segregation in foraging niche is importantly mediated by SSD, and b) sex differences in SI signatures were much more common during the pre-laying and breeding season, when competition is greater due to reduced foraging ranges, than during the non-breeding season. The latter finding lends support to an important hypothesis of sexual segregation in animals: dominant individuals, i.e., larger individuals, often males, out-compete subordinate conspecifics, i.e., smaller or less experienced individuals, often females or juveniles, for high quality habitat [23], [24].

**Figure 1:**
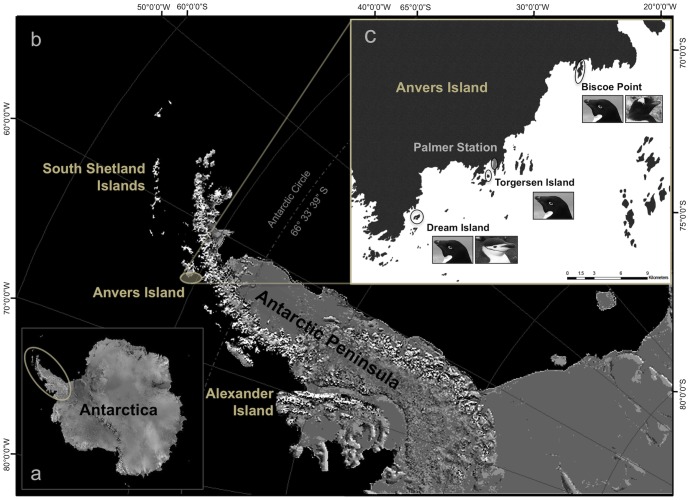
The marine ecosystem west of the Antarctic Peninsula (a) extends from northern Alexander Island to the South Shetland Islands (b). Fieldwork for the present study took place within the Palmer Archipelago near Anvers Island and Palmer Station, a United States supported research base (b, c). Penguin rookeries were located at Dream [Adélie and chinstrap], Torgersen [Adélie only], and Biscoe [Adélie and gentoo] Islands (c). Image generated from base maps provided by the National Snow and Ice Data Center's map server A-CAP (http://nsidc.org/agdc/acap/).

*Pygoscelis* penguins, see [25] for Spheniscidae phylogeny, occurring throughout the marine ecosystem west of the Antarctic Peninsula (AP, Fig. 1a, b) provide a unique system for testing the hypothesis that ecological sexual dimorphism increases or decreases depending on levels of competition for nutritional resources, following [19]. Changes in environmental conditions that affect the quality and/or quantity of prey might be an important driver of variation in foraging competition between males and females, however such variability has not been extensively examined in the context of ecological sexual dimorphism among penguins, but see [26]. We explore this issue within an interesting community context in which winter environmental conditions would be expected to result in differing inter-specific levels of foraging competition between males and females given generally opposing life history affinities among species for Southern Ocean sea ice, a key physical parameter structuring polar marine food-webs [27], [28].

Sea ice is a critical ecological feature structuring habitat use by *Pygoscelis* penguins. Adélie penguins (*P*. *adeliae*) are associated with pack ice habitat during winter [29]–[32], while chinstrap penguins (*P. antarctica*) are noted to winter off-shore, in sea ice-free waters predominantly throughout the northwestern AP and Scotia Sea [33], [34]. Gentoo penguins are noted to inhabit near shore, open water wintering areas close to breeding colonies [35]. During the summer breeding season, Adélie penguins hold a circumpolar distribution at high southern latitudes, nesting within terrestrial rookeries located in close proximity to marine foraging areas generally characterized by persistent summer sea ice [29]. Only along the northwestern AP region do Adélie penguins breed in sympatry with their sea ice-intolerant congeners. Although Adélie penguins initiate nesting earlier here [36], all three species typically forage in sea ice-free waters during summer as a result of marked ocean-climate warming [36], [37] and associated reductions in annual sea ice coverage [38], [39], particularly within the northern seasonal sea ice zone [40], see also [41] Figure 1. Adélie penguins breeding within the northwestern AP are considered mismatched with current environmental conditions given notable population declines [36], [42]–[45]. Therefore, *Pygoscelis* penguins appear to hold opposing life-history affinities for the presence of sea ice [29], [33]–[35], [42] that have been shaped over evolutionary time scales [25]. However, associated trophic pathways critical to each species, and especially each sex within species, have not been well resolved.

Here, we examined ecological sexual dimorphism among adult *Pygoscelis* penguins nesting within the northwestern AP, along the Palmer Archipelago located near Anvers Island (Fig. 1b, c). Specifically, we considered variation in δ^13^C and δ^15^N SI signatures of blood tissue, obtained during egg laying, as a biogeochemical proxy of pre-breeding trophic foraging given that these isotopes are known to reliably reflect trophic position [46], [47] by integrating dietary information over approximately the previous 30 to 60 days given allometric turn-over rates for the cellular fraction of blood [18], [48]–[50]. We predicted that males and females of all three species should show sex-specific foraging given that male and female gentoo penguins nesting at Bird Island, South Georgia, have been shown to differ in their breeding foraging niche [18] and hold generally similar, moderate levels of SSD as Adélie and chinstrap penguins [51], [52]. We also predicted that in years of relatively higher quality winter habitat, i.e., greater sea ice coverage for Adélie penguins and lower coverage for chinstrap and gentoo penguins, males and females would be more similar in their pre-breeding foraging niche given reduced competition for prey as a test of the conclusion by Phillips et al. [19].

We first (1) validated molecular primers for universal sex determination of all three *Pygoscelis* species. In birds, the chromobox-helicase-DNA-binding (CHD) gene CHD-Z occurs in both males (ZZ) and females (ZW), while CHD-W only occurs in females. By amplifying primers specific to both genes from a sample of genomic DNA, gel electrophoresis is expected to reveal two bands in females and one band in males. Thus, we tested two sets of primers, P2/P8 and 2550F/2718R, for consistent amplification of CHD-Z and CHD-W regions [53], [54] using mated adults at the one-egg stage where each pair is expected to include one male and one female. Based on our molecular sexing data, we (2) quantified levels of adult SSD within each species by developing predictive models of sex based on field-measured morphometrics of breeding adults, and then tested the predictive utility of best supported morphometric models for determining sex, using an independent dataset. We (3) calculated a commonly used index of SSD following Lovich and Gibbons [55] to quantify structural size for each species based on morphometric parameters considered in previous analyses. Lastly, we (4) asked whether SSD-associated sex (as defined previously), or structural size independent of sex, are better predictors of pre-breeding isotopic foraging niche while also assessing support for the hypothesis that yearly environmental variability, discussed within the context of winter sea ice conditions, interacts with sex- or size-specific foraging.

## Materials and Methods

### Ethics statement

Research was conducted in accordance with an Antarctic Conservation Act permit to WRF (2008-020), in addition to Canadian Committee on Animal Care guidelines (Simon Fraser University, SFU, Animal Care Permit 890B-08 to KBG and TDW).

### Field methods

Field research was conducted on *Pygoscelis* penguins nesting on several islands within the Palmer Archipelago west of the AP near Anvers Island (64°46′S, 64°03′W, Fig. 1a-c), during the austral summers of 2007/08, 2008/09, and 2009/10. Specifically, study nests were located on Biscoe (64°48′S, 63°46′W), Torgersen (64°46′S, 64°04′W), and Dream (64°43′S, 64°13′W) Islands (Fig. 1c). Each study season, Adélie penguin study nests (*n* = 30) were distributed equally between the three study islands, with 10 nests located on each island. Gentoo penguin study nests (*n* = 30) were all located on Biscoe Island, while chinstrap penguin study nests (*n* = 15) were all located on Dream Island (Fig. 1c). The reduced sample size for chinstraps was due to the overall smaller number of individuals breeding at rookeries on Dream Island.

Each season, study nests, where pairs of adults were present, were individually marked and chosen before the onset of egg laying, and consistently monitored. When study nests were found at the one-egg stage, both adults were captured to obtain blood samples used for molecular sexing and SI analyses, and measurements of structural size and body mass. At the time of capture, each adult penguin was quickly blood sampled (∼1 ml) from the brachial vein using a sterile 3 ml syringe and heparinized infusion needle. Collected blood was stored in 1.5 ml micro-centrifuge tubes that were kept cool. In the field, a small amount of whole blood was smeared on clean filter paper stored in a 1.5 ml micro-centrifuge tube for molecular sexing. Measurements of culmen length and depth (using dial calipers ±0.1 mm), right flipper (using a ruler ±1 mm), and body mass (using 5 kg±25 g or 10 kg±50 g Pesola spring scales and a weigh bag) were obtained to quantify body size variation. After handling, individuals at study nests were further monitored to ensure the pair reached clutch completion, i.e., two eggs.

**Figure 2:**
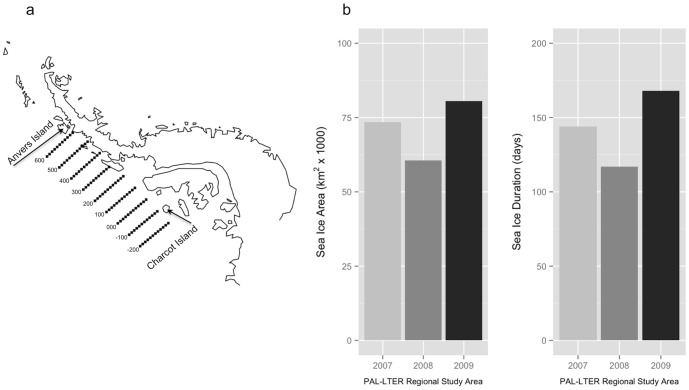
Palmer Station, Antarctica, Long Term Ecological Research (PAL-LTER) Program's regional study area west of the Antarctic Peninsula, extending 800 km from Anvers Island (600 line) to just south of Charcot Island (−200 line) and 220 km inshore to offshore (a). Study area grid lines encompass the entire area over which sea ice metrics were calculated. Winter average sea ice area (km^2^) and sea ice duration (days) within the PAL-LTER regional study area for each year of the present study (b).

Sea ice data were based on satellite estimates of sea ice concentration (SIC) within the Palmer Station, Antarctica, Long-Term Ecological Research (PAL-LTER) Program's regional study grid (Figs. 1, 2a). Daily and monthly time series of SIC were used to calculate two metrics, 1-average winter sea ice area (km^2^) representing the yearly average area covered by winter sea ice, and 2-duration (days) representing the yearly total length of the winter sea ice season, both within the PAL-LTER grid, using time series based on Version 2 of the Goddard Space Flight Center (GSFC) Bootstrap algorithm [56] provided by the National Snow and Ice Data Center (NSIDC; www.nsidc.org). These sea ice metrics were derived based on 1-monthly averages between the winter months of sea ice advance and retreat, and 2-the day of first and last appearance of sea ice within the same region and averaged for the PAL-LTER grid. Both metrics are useful for describing both the winter spatial and temporal variability of Southern Ocean sea ice [38].

### Laboratory methods

Within 12 hours (hrs) of field collection, tubes containing whole blood were centrifuged to separate plasma and red blood cell (RBC) fractions, which were stored separately and frozen at −80 degrees Celsius (°C). Tubes containing whole blood smears on filter paper were allowed to dry in a desiccator. After drying, tubes were sealed and frozen at −80°C.

Tubes containing RBCs were first allowed to dry to a consistent mass in a drying oven at 60°C. Using a mortar and pestle lined with clean weighing paper, dried RBC pellets were homogenized into a powder. Each mortar and pestle was washed and dried in between sample processing. Aliquots of powdered samples were transferred to 8×5 mm pressed tin capsules (Elemental Microanalysis) and weighed (∼2 mg) using an analytical balance. Samples were organized in 96-microwell plates and analyzed for δ^13^C and δ^15^N SI signatures using an elemental analyzer interfaced with an isotope ratio mass spectrometer at the Stable Isotope Facility, University of California (UC) - Davis. Data expressed as δ^13^C or δ^15^N were calculated using the following equation: δ^13^C or δ^15^N = ([R_sample_/R_standard_]-1)×1000, where R_sample_ is the ratio of the heavy to light isotope for either ^13^C/^12^C or ^15^N/^14^N, and R_standard_ is the heavy to light isotope ratios for international standards - Vienna PeeDee Belemnite for carbon, and atmospheric N_2_ (Air) for nitrogen.

Whole blood smears were allowed to dry a second time in a desiccator for at least 24 hrs prior to analysis. Sex of adult *Pygoscelis* penguins was determined molecularly using PCR amplification as outlined by Griffiths et al. [53], as well as Fridolfsson and Ellegren [54]. See Supporting Information Text S1 for specific details regarding PCR methods including extraction, amplification, and gel electrophoresis.

### Statistical methods

Percent of mated adult pairs correctly identified by the primers P2/P8 and 2550F/2718R was calculated for a subset of individuals. Correct identification was defined as gel electrophoresis revealing one female and one male, per pair. DNA from five adult pairs per species (*n* = 30 individuals total) for each primer set was used in the analysis.

Final sample sizes for study nests/individual adults of each species were *n* = 76/152 for Adélie, *n* = 34/68 for chinstrap, and *n* = 62/124 for gentoo penguins over the three years of study. These sample sizes are reduced in comparison with the original number of study nests marked and monitored per species as at times weather conditions hindered rookery access resulting in some study nests not being sampled if the pair had already reached clutch completion. In addition, of those adults sampled, some pairs were excluded from statistical analyses because a final egg was never observed at the nest, likely due to brown skua (*Stercorarius lonnbergi*) depredation. Therefore, it was unknown whether these individuals represented adults exactly at the one-egg stage. All statistical analyses were performed in the R language environment [57].

To assess the explanatory value of various structural parameters for discerning sex of adult penguins, logistic regression was employed using generalized linear models (GLM, family  =  binomial) to account for a response where males were coded as 0 and females coded as 1, in relation to four continuous structural size parameters including 1-culmen depth, 2-culmen length, 3-flipper length, and 4-body mass. Information-theoretic methods, performed in R, were used to direct model selection and parameter estimation following Burnham and Anderson [58]. An *a priori* set of 15 candidate models consisted of an equal-means (null) model and all combinations of culmen depth and length, flipper length, and body mass as main effects (14 models). This same candidate model set was evaluated for all three species, separately, using a truncated dataset (2/3^rds^) of randomly chosen individuals (Adélie *n* = 88, chinstrap *n* = 36, gentoo *n* = 74). For each species' analysis, we first assessed overdispersion (ĉ) in the most parameterized model using the following equation: ĉ = residual deviance/residual degrees of freedom, see [58] page 68. In the event ĉ was found to be greater than 1, quasi Akaike's Information Criterion (QAICc) values, which include a correction for small sample size, would be calculated for each model to correct for overdispersion. Where ĉ was found to be less than 1, Akaike's Information Criterion (AICc) values, again corrected for small sample size, would be calculated for each model. In addition, ΔAICc and Akaike weight (*w*) values were calculated and used to compare candidate models [58]. For each candidate model, a pseudo *r* ^2^mf (McFadden) value was calculated to provide a measure of fit for each model, where *r* ^2^mf = 1-(logLikelihood(model)/logLikelihood(null model)) [59]. Inference was based on the relative support for parameters across all models and weighted parameter estimates. Parameter estimation included calculation of model-averaged parameter estimates based on *w* values for all candidate models. Standard errors (SE) for parameter estimates were based on unconditional variances calculated across the same models. Parameter likelihood values were evaluated by summing *w* values across all models that included each parameter under consideration [58].

To assess the predictive utility of these same structural measurements to determine sex of adult penguins, we calculated the probability of individuals being female (1), using the *predict* function in R, with a dataset (1/3^rd^) comprised of individuals (Adélie *n* = 44, chinstrap *n* = 18, gentoo *n* = 38) not included in developing most parsimonious structural size models as described above. Models used to predict sex were those best supported, in analyses described above, which received ΔAICc values ≤2.

To quantify levels of SSD in adults of each species, we calculated a standard index of size dimorphism (SDI) following Lovich and Gibbons [55], where SDI  =  ((mean size of larger sex/mean size of smaller sex) - 1) with the resulting value arbitrarily defined as positive when females are larger and negative when males are larger (Adélie *n* = 137, chinstrap *n* = 54, gentoo *n* = 116). An SDI was calculated for all structural parameters considered as best predictors of adult penguin sex for each species. In addition, two overall average SDIs were calculated based on SDIs of 1-all four structural parameters, and 2-culmen depth, length and flipper length SDIs only.

Least-squares general linear models (LM) were used to examine continuous variation in δ^13^C and δ^15^N SI signatures of adult penguin RBCs in relation to three parameters treated as main effects including 1-sex, as determined by molecular data and treated categorically, 2-overall size using a principal components score (PC1) based on culmen depth and length, and flipper length and treated continuously, and 3-year, treated categorically. Individual scores for PC1 were calculated using the *prcomp* function in R for datasets consisting of both males and females, but calculated separately for each species. An *a priori* set of eight candidate models consisted of an equal-means model, each predictor variable as a main effect (three models), additive models for sex or size with year, defined so that sex and size were never included in the same model (two models), and interaction models for these same additive models where an interaction was included for each parameter considered as a main effect in the model (two models). This same candidate model set was evaluated for each isotope separately using datasets for each species (Adélie *n* = 127 for δ^13^C and *n* = 128 for δ^15^N due to the exclusion of one data point as a δ^13^C outlier based on residual plots for normality, chinstrap *n* = 53, gentoo *n* = 115). For each candidate model, AICc, ΔAICc and *w* values were calculated and used to compare models [58], as well as an *r* ^2^ value, defined as the fraction of the total variance explained by the model and given by the Multiple R-squared calculation in R, see [60] page 399, to provide a general measure of fit for each model. Inference was based on model averaging with parameter estimation following that described above for logistic regression analyses.

### Data management

Data reported here are publicly available within the PAL-LTER data system (datasets #219, 220, and 221): http://oceaninformatics.ucsd.edu/datazoo/data/pallter/datasets. These data are additionally archived within the United States (US) LTER Network's Information System Data Portal: https://portal.lternet.edu/. Individuals interested in using these data are therefore expected to follow the US LTER Network's Data Access Policy, Requirements and Use Agreement: http://www.lternet.edu/policies/data-access.

## Results

### Primer validation

P2/P8 primers correctly identified 100% of each species' pairs, while 2550F/2718R primers correctly identified 60% of Adélie and gentoo penguin pairs and 0% of chinstrap penguin pairs. See Supporting Information Figure S1 for an image of PCR bands from both primer sets and Supporting Information Text S2 for details on PCR optimization with P2/P8 primers. Of the entire dataset for each species, P2/P8 primers failed to amplify in one of 147 adult Adélie penguins and four of 123 adult gentoo penguins. P2/P8 primers amplified in all 68 adult chinstrap penguins.

### Quantifying SSD

Overdispersion (ĉ) was found to be less than 1 for each species analysis (Adélie ĉ = 0.37, chinstrap ĉ = 0.44, gentoo ĉ = 0.23), precluding the need to calculate QAICc values for each model. For Adélie penguins, three models received ΔAICc values ≤2 for predicting sex from structural parameters. The most parsimonious model received a slightly higher *w* value (0.39) than the second and third best supported model, which received similar values for *w* (0.31 and 0.30, respectively). All supported models received high *r* ^2^mf values (>70%) with the best supported model including terms for culmen length and depth, as well as body mass. The second supported model included only terms for culmen length and body mass. However, the global model including all parameters was the third best supported model (Table 1). Parameter likelihood values indicated very strong support for culmen length and body mass, moderately strong support for culmen depth, but much lower support for flipper length given the data and candidate model set (Table 2).

**Table 1:** Candidate models for predicting sex of *Pygoscelis* penguins.

Species	Response variable	Model number	Explanatory variable	Number of parameters	ΔAICc	*w*	*r* ^2^mf	% correctly classified
Adélie penguin	Sex	1	Culmen length + Culmen depth + Body mass	4	0.000	0.392	0.73	88.64%
		2	Culmen length + Body mass	3	0.480	0.308	0.71	88.64%
		3	Culmen length + Culmen depth + Flipper length + Body mass	5	0.539	0.299	0.75	88.64%
Chinstrap penguin	Sex	1	Culmen length + Culmen depth	3	0.000	0.37	0.70	94.44%
Gentoo penguin	Sex	1	Culmen length + Culmen depth + Body mass	4	0.000	0.528	0.85	89.47%

Models presented are those determined to be most parsimonious, as well as all models receiving ΔAICc values ≤2. Percent of individuals from independent datasets correctly classified for sex based on probability estimates using supported models.

Abbreviations: ΔAICc  =  Akaike's Information Criterion corrected for small sample size, *w*  =  Akaike weight, *r* ^2^mf  =  pseudo *r* ^2^ McFadden.

**Table 2:** Parameter estimates and likelihoods from candidate models for predicting sex of *Pygoscelis* penguins.

Species	Response variable	Explanatory variable	Parameter likelihood	Parameter estimate (±1 SE)	Size Dimorphism Index
Adélie penguin	Sex	Intercept	1.000	77.00±23.38	
		Culmen length	0.999	−1.04±0.34	−0.09
		Culmen depth	0.691	−0.58±0.41	−0.08
		Flipper length	0.299	0.03±0.03	−0.02
		Body mass	0.999	−0.009±0.003	−0.20
					AVE -0.10 (Overall)
					AVE -0.06 (-Body mass)
Chinstrap penguin	Sex	Intercept	1.000	88.86±37.16	
		Culmen length	0.816	−0.687±0.37	−0.11
		Culmen depth	0.841	−2.01±1.07	−0.10
		Flipper length	0.407	−0.12±0.13	−0.05
		Body mass	0.328	0.002±0.002	−0.10
					AVE -0.09 (Overall)
					AVE -0.09 (-Body mass)
Gentoo penguin	Sex	Intercept	1.000	138.78±57.35	
		Culmen length	0.739	−0.72 ±0.46	−0.08
		Culmen depth	0.954	−3.05±1.50	−0.10
		Flipper length	0.299	−0.02±0.08	−0.04
		Body mass	0.999	−0.01±0.004	−0.17
					AVE -0.10 (Overall)
					AVE -0.07 (-Body mass)

Parameter estimates (±1 SE) are weighted averages, and standard errors are based on unconditional variances. Parameter likelihoods are Akaike weight (*w*) values summed across all models that include the variable. Size dimorphism indices are also reported for each structural parameter.

Abbreviations: ± 1SE  =  plus or minus 1 standard error, AVE  =  average.

For chinstrap penguins, one model received ΔAICc values ≤2. The most parsimonious model received a higher *w* value (0.37) than the second ranked model (*w* = 0.12), a high *r* ^2^mf value (70%), and included terms for culmen length and depth (Table 1). Parameter likelihood values indicated strong support for culmen length and depth, but weaker support for flipper length and body mass given the data and candidate model set (Table 2).

For gentoo penguins, one model received ΔAICc values ≤2. The most parsimonious model received a higher *w* value than the next best supported model (0.53 and 0.17, respectively) and a high *r* ^2^mf value (85%). The best supported model included terms for culmen length and depth, and body mass (Table 1). Parameter likelihood values indicated very strong support for culmen depth and body mass, and moderately strong support for culmen length, but only weak support for flipper length given the data and candidate model set (Table 2).

Probability estimates of adult Adélie penguin sex, based on models receiving ΔAICc values ≤2 as reported above and tested on independent datasets, suggested that when using 0.50 as a probability threshold (i.e., >0.50 =  female, <0.50 = male), all supported models accurately predicted sex for 39 out of 44 individuals (88.64%, Table 1). For chinstrap penguins, the most parsimonious model accurately classified 94.44% individuals (Table 1). For gentoo penguins, the most parsimonious model accurately predicted sex for 34 out of 38 individuals (89.47%, Table 1). See Supporting Information Text S3 for details on probability estimates and misclassification.

As expected, *Pygoscelis* penguin SDIs were larger for morphological parameters that better predicted sex of adult penguins (Table 2). Negative SDI values indicate that for each structural parameter male *Pygoscelis* penguins were, on average, larger than females.

### Environmental variability and foraging niche

Sea ice metrics indicated that the austral winters preceding each study year were generally characterized by lower than average sea ice coverage, with winter 2008 being the lowest sea ice season in the PAL-LTER record since 1979 [38], see also sea ice datasets within the PAL-LTER data system. Relative yearly variability in sea ice (Fig. 2b) over the course of the study suggested that 2007 was an intermediate sea ice season relative to 2008 (low) and 2009 (high). Results for inter-annual variability in foraging niche of *Pygoscelis* penguins are discussed within the context of these qualitative differences in each year's sea ice season.

**Figure 3:**
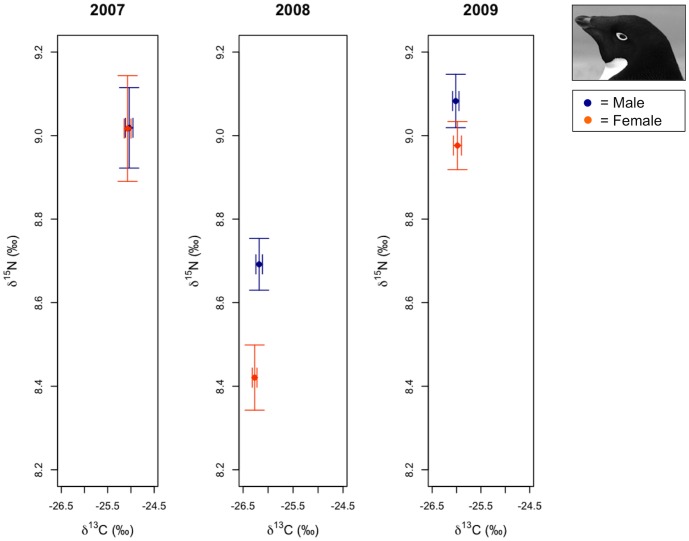
Variation in Adélie penguin δ^13^C and δ^15^N stable isotope signatures. Average male and female isotope signatures (± 1 SE) are presented for each year based on sample sizes exactly following analyses (*n* = 127 for δ^13^C and *n* = 128 for δ^15^N).

Scores for PC1 explained 56%, 71% and 78% of the total variance in body size for Adélie, chinstrap and gentoo penguins, respectively. Adélie penguin analyses resulted in two models receiving ΔAICc values ≤2 for predicting variation in δ^13^C SI signatures. The most parsimonious model, which included the year term only, received a considerably higher *w* value (0.53) than that for the next best supported model (*w* = 0.21), which included terms for sex and year. Both models had high *r* ^2^ values (0.71) (Table 3). Parameter likelihoods indicated strong support for the year term, but only weak support for the sex term given the data and the candidate model set (Table 4, Fig. 3). PC1 and the sex term received similar parameter likelihoods as the model including both PC1 and Year was the third ranked model. However, both models with either sex or PC1 terms only were ranked lower than the equal-means model, suggesting that these parameters provided little explanatory value. No other explanatory variables, such as any of the interaction terms, were strongly supported by the data (Table 4). Inter-annual variability in δ^13^C SI signatures of Adélie penguins did not associate with qualitative variation in winter sea ice conditions given that weighted parameter estimates for both 2008 (−1.17±0.14 95% confidence interval, CI  =  parameter estimate SE*1.96) and 2009 (−0.94±0.14 CI), were depleted in years with the lowest and highest sea ice conditions during the study (Table 4, Fig. 2b). Two models also received ΔAICc values ≤2 for predicting variation in Adélie penguin δ^15^N SI signatures. The most parsimonious model, which included terms for sex and year, received a higher *w* value (0.53) then that for the next best supported model (*w* = 0.25), which included these same parameters as main effects and an interaction for sex*year. Both models had similar *r* ^2^ values (0.30 and 0.32, respectively) (Table 3). Parameter likelihoods suggested stronger support for sex and year terms, however, the interaction term for sex*year received less support given the data and the candidate model set (Table 4, Fig. 3). Weighted parameter estimates for the year term indicated that Adélie penguin δ^15^N SI signatures were depleted in 2008 (−0.43±0.21 CI) relative to higher sea ice years in 2007 and 2009 (0.02±0.19 CI; Table 4, Fig. 3). Weighted parameter estimates suggested that female Adélie penguin δ^15^N SI signatures were not depleted relative to males (−0.08±0.17 CI). Confidence intervals overlapped 0 indicating that sex was not important in accounting for variation in Adélie δ^15^N SI signatures (Table, 4, Fig. 3). Weighted parameter estimates for the interaction between sex*year suggested a possibly larger effect in 2008, the year with low sea ice (−0.07±0.13 CI), than in 2009 (−0.03±0.09 CI), however confidence intervals for both years overlapped 0, indicating that this interaction also was not important in accounting for variation in Adélie δ^15^N SI signatures (Table, 4, Fig. 3).

**Table 3:** Candidate models for describing variation in foraging niche of *Pygoscelis* penguins assessed by δ^13^C and δ^15^N stable isotope signatures.

Species	Response variable	Model number	Explanatory variable	Number of parameters	ΔAICc	*w*	*r* ^2^
Adélie penguin	δ^13^C	1	Year	4	0.000	0.530	0.71
		2	Sex + Year	5	1.858	0.209	0.71
Chinstrap penguin	δ^13^C	1	Equal-means model	2	0.000	0.286	0.00
		2	Year	4	0.484	0.225	0.08
		3	PC1	3	1.379	0.144	0.02
		4	Sex	3	1.553	0.132	0.01
Gentoo penguin	δ^13^C	1	Sex + Year	5	0.000	0.428	0.94
		2	PC1 + Year	5	0.903	0.272	0.94
		3	Year	4	1.665	0.186	0.94
Adélie penguin	δ^15^N	1	Sex + Year	5	0.000	0.528	0.30
		2	Sex + Year + Sex*Year	7	1.489	0.251	0.32
Chinstrap penguin	δ^15^N		Sex + Year	5	0.000	0.884	0.53
Gentoo penguin	δ^15^N	1	Sex + Year	5	0.000	0.578	0.50
		2	Sex + Year + Sex*Year	7	1.346	0.295	0.51

Models presented are those determined to be most parsimonious, as well as all models receiving ΔAICc values ≤2.

Abbreviations: ΔAICc  =  Akaike's Information Criterion corrected for small sample size, *w*  =  Akaike weight, *r* ^2^mf  =  pseudo *r* ^2^ McFadden.

**Table 4:** Parameter estimates and likelihoods from candidate models for describing variation in foraging niche of *Pygoscelis* penguins assessed by δ^13^C and δ^15^N stable isotope signatures.

Species	Response variable	Explanatory variable	Parameter likelihood		Parameter estimate ±1 SE	
			δ^13^C	δ^15^N	δ^13^C	δ^15^N
Adélie penguin	δ^13^C or δ^15^N	Intercept	1.000	1.000	−25.05±0.06	9.06±0.08
		Sex	0.245	0.778	−0.008±0.02	−0.08±0.09
		PC1	0.227	0.077	0.0005±0.006	−0.002±0.004
		Year 2008	0.999	0.999	−1.17±0.07	−0.43±0.11
		Year 2009	0.999	0.999	−0.95±0.07	0.02±0.01
		Sex*Year 2008	0.037	0.251	−0.002±0.006	−0.07±0.07
		Sex*Year 2009	0.037	0.251	0.003±0.006	−0.03±0.05
		PC1*Year 2008	0.048	0.027	0.0006±0.003	0.003±0.003
		PC1*Year 2009	0.048	0.027	−0.003±0.004	0.002±0.003
Chinstrap penguin	δ^13^C or δ^15^N	Intercept	1.000	1.000	−24.57±0.04	9.12±0.08
		Sex	0.243	0.963	−0.01±0.02	−0.22±0.08
		PC1	0.247	0.017	0.004±0.006	0.0006±0.0008
		Year 2008	0.439	0.999	−0.01±0.03	0.38±0.10
		Year 2009	0.439	0.999	0.04±0.04	0.57±0.09
		Sex*Year 2008	0.0104	0.078	−0.0003±0.002	−0.008±0.02
		Sex*Year 2009	0.010	0.078	0.0008±0.002	−0.004±0.01
		PC1*Year 2008	0.011	0.002	−0.0004±0.0007	5.83E−05±0.0001
		PC1*Year 2009	0.011	0.002	−0.0002±0.0006	8.76E−05±0.0001
Gentoo penguin	δ^13^C or δ^15^N	Intercept	1.000	1.000	−25.42±0.03	8.02±0.05
		Sex	0.493	0.872	−0.03±0.02	−0.11±0.05
		PC1	0.321	0.107	0.004±0.005	0.003±0.003
		Year 2008	0.999	0.999	−1.32±0.03	0.43±0.06
		Year 2009	0.999	0.999	−0.72±0.03	0.30±0.05
		Sex*Year 2008	0.066	0.295	0.003±0.005	0.04±0.04
		Sex*Year 2009	0.066	0.295	−0.0003±0.004	0.01±0.03
		PC1*Year 2008	0.049	0.014	0.0006±0.001	−0.0004±0.0006
		PC1*Year 2009	0.049	0.014	0.001±0.002	−0.0001±0.0005

Parameter estimates (±1 SE) are weighted averages, and standard errors are based on unconditional variances. Parameter likelihoods are Akaike weight (*w*) values summed across all models that include the variable.

Abbreviations: ± 1SE  =  plus or minus 1 standard error.

**Figure 4:**
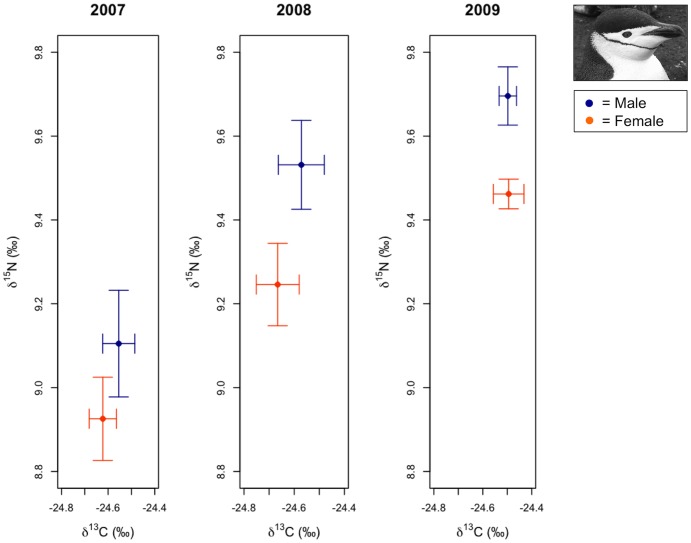
Variation in chinstrap penguin δ^13^C and δ^15^N stable isotope signatures. Average male and female isotope signatures (± 1 SE) are presented for each year based on sample sizes exactly following analyses (*n* = 53).

Chinstrap penguin analyses resulted in four models receiving ΔAICc values ≤2 for predicting variation in δ^13^C SI signatures, however, the best supported model was the equal-means model (Table 3). Parameter likelihoods indicated little support for all explanatory parameters including interaction terms (Table 4). Variation in chinstrap δ^13^C SI signatures was not qualitatively associated with winter sea ice conditions (Fig. 4). One model received ΔAICc values ≤2 for predicting variation in chinstrap penguin δ^15^N SI signatures, which included terms for sex and year as main effects only. This model received a high *w* value (0.88) and a moderately high *r* ^2^ value (0.53) (Table 3). Accordingly, sex and year terms received strong support based on parameter likelihoods, while all other parameters received essentially no support given the data and candidate model set (Table 4, Fig. 4). Weighted parameter estimates for the year term indicated that chinstrap penguin δ^15^N SI signatures were enriched in 2008 (0.38±0.19 CI) and 2009 (0.57±0.17 CI) suggesting no obvious relationship with variation in winter sea ice conditions (Table 4, Fig. 4). Weighted parameter estimates for the sex term indicated that female chinstrap penguins (−0.22±0.15 CI) were depleted in δ^15^N relative to males (Table 4, Fig. 4).

**Figure 5:**
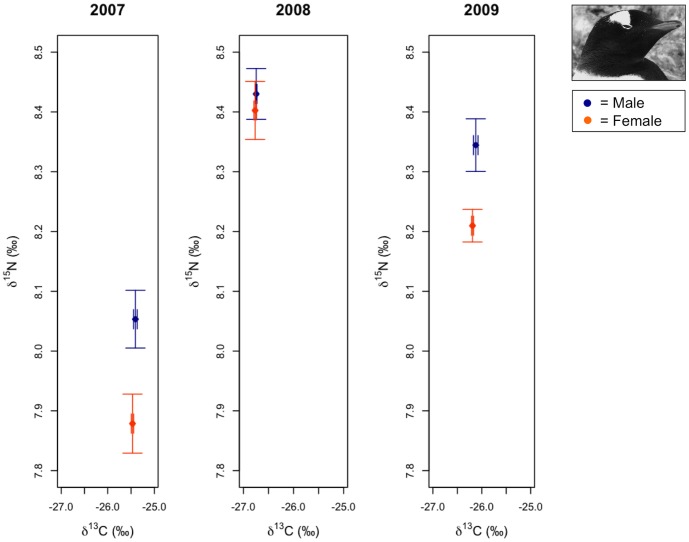
Variation in gentoo penguin δ^13^C and δ^15^N stable isotope signatures. Average male and female isotope signatures (± 1 SE) are presented for each year based on sample sizes exactly following analyses (*n* = 115).

Gentoo penguin analyses resulted in three models receiving ΔAICc values ≤2 for predicting variation in δ^13^C SI signatures. The most parsimonious model included terms for sex and year, which received a higher *w* value (0.43) then the next best supported model that included terms for PC1 and year (*w* = 0.27). However, both models had very high *r* ^2^ values (0.94). The third ranked model included the year term only, and received a low *w* value (0.19), but also a very high *r* ^2^ value (0.94, Table 3). Parameter likelihoods indicated strong support for the year term, and moderate support for the sex term given the data and the candidate model set (Table 4, Fig. 5). The PC1 term received a slightly lower parameter likelihood than the sex term as the model including both PC1 and year was the second ranked model. However, both models with either sex or PC1 terms only were ranked lower than the equal-means model, suggesting that these parameters provided little explanatory value. No other explanatory variables, such as any of the interaction terms, were strongly supported by the data (Table 4). Weighted parameter estimates for the year term indicated that gentoo penguin δ^13^C SI signatures were depleted in 2008 (−1.33±0.07 CI) and 2009 (−0.72±0.07 CI), the years with lowest and highest sea ice conditions, respectively (Table 4, Fig. 5). Weighted parameter estimates for sex (−0.03±0.04 CI) and PC1 score (0.004±0.009 CI) both overlapped 0 indicating these variables were not important in explaining variation in δ^13^C SI signatures of gentoo penguins (Table 4, Fig. 5). Two models received ΔAICc values ≤2 for predicting variation in gentoo penguin δ^15^N SI signatures. The most parsimonious model, which included terms for sex and year, received a higher *w* value (0.58) than that for the next best supported model (*w* = 0.30), which included these same parameters as main effects and an interaction for sex*year. Both models had similar *r* ^2^ values (0.50 and 0.51, respectively) (Table 3). Parameter likelihoods suggested stronger support for sex and year terms, however, the interaction term for sex*year received less support given the data and the candidate model set (Table 4, Fig. 5). Weighted parameter estimates for the year term indicated that gentoo penguin δ^15^N SI signatures were most enriched in 2008 (0.43±0.12 CI) and slightly less enriched in 2009 (0.30±0.10 CI), years with the greatest difference in sea ice conditions (Table 4, Fig. 5). Weighted parameter estimates for the sex term indicated that gentoo penguin δ^15^N SI signatures were depleted in females (−0.11±0.09 CI) relative to males (Table 4, Fig. 5). Lastly, weighted parameter estimates for the interaction between sex*year (2008: 0.04±0.08 CI, 2009: 0.01±0.06 CI) suggested that this interaction was not important in accounting for variation in gentoo δ^15^N SI signatures as confidence intervals for both years overlapped 0 (Table, 4, Fig. 5).

## Discussion

Our primer validation adds to a growing body of literature that has used molecular techniques for gender determination of penguins [61]–[66], however, much of this work was aimed at developing morphometric methodologies for sex determination. We used molecular sexing data to quantify SSD specifically among *Pygoscelis* penguins nesting along the Palmer Archipelago given that structural size of penguins can vary regionally [52], and therefore, SSD indices developed from other populations may not be readily applicable to our own study system. We further demonstrated that overall larger size dimorphism indices between species were associated with greater differences in sex-specific pre-breeding foraging niche based on δ^15^N SI signatures of RBCs. However, there was no strong evidence for the hypothesis that ecological sexual dimorphism increased, or decreased, in association with higher or lower quality winter habitat that might induce variation in foraging competition between males and females.

The molecular primers P2/P8 were more reliable genetic markers for determining sex of all three *Pygoscelis* species than 2550F/2718R primers; P2/P8 successfully amplified in 99.3% of Adélie penguins, 100% of chinstrap penguins, and 96.8% of gentoo penguins sampled from our study population. Hart et al. [64] showed P2/P8 primers to have a much higher rate of successful amplification than 2550/2718 primers in macaroni penguins (*Eudyptes chrysolophus*). A recent paper by Polito et al. [66] used P0/P2/P8 primers to successfully sex 94% of *Pygoscelis* penguins, i.e., 97 of 103 individuals tested, nesting at Admiralty Bay, King George Island, Antarctica. Our slightly higher success rate of P2/P8 amplification in *Pygoscelis* penguins may be due to either a) differing quality of genomic material offered by whole blood versus tissue from the feather calamus, or b) the different thermal profiles used by each study, compared to [66].

Estimates of penguin SSD have generally relied on one or only a few morphological features. Williams [67] used bill length and depth to assess structural size differences between male and female gentoo penguins. Fairbairn and Shine [51] relied on published estimates of body mass for 17 species of Spheniscidae in their meta-analysis of seabird SSD, while Polito et al. [66] considered measurements of bill features only for developing SSD indices for *Pygoscelis* penguins. The evolution of SSD is primarily considered a result of sexual and/or fecundity selection [6], therefore, it is highly likely that several traits are under selection that result in overall body size differences between the sexes. To this end, we considered a suite of morphological features in our analyses similar to Bertellotti et al. [61], to more broadly assess SSD in *Pygoscelis* penguins. We recognize that body mass is a plastic trait that can vary over the annual cycle, therefore, our body mass results are truly only relevant for individuals during the egg laying period and should not be considered representative of individuals during other seasonal phases such as chick rearing or outside the breeding season.

Our results demonstrate inter-specific differences in best morphological predictors of sex among *Pygoscelis* penguins. Adélie penguin body mass and culmen length were the strongest predictors of sex, while body mass and culmen depth were best predictors of male and female gentoo penguins. For chinstrap penguins, body mass was the least predictive structural feature, while culmen length and depth were similarly strong predictors of sex. Species-specific models based on these best morphological predictors correctly classified a high percentage of individuals from independent datasets (i.e., 89–94%). Interestingly, flipper length was not a strong predictor of sex for any of the three species. Culmen features and body mass are structures important during penguin courtship [52], and therefore, likely targets of sexual selection, which may be why these parameters are strong predictors of sex across *Pygoscelis* species. Similar results were found by Bertellotti et al. [61] who included bill depth and length, but not flipper length, of Magellanic penguins (*Spheniscus magellanicus*) in a discriminant function that correctly classified 97% of adults.

Variation in our calculated size dimorphism indices generally reflected differences in best morphological predictors of adult penguin sex discussed above, which would be expected. Size dimorphism indices for Adélie penguin body mass and culmen length were the largest of those calculated for the species. Similarly, SDIs for gentoo penguin body mass and culmen depth were the largest calculated. Culmen length and depth of chinstrap penguins were equally strong predictors of sex, but associated SDIs were similar to the index for body mass, which was the least predictive structural feature for male and female chinstrap penguins. The overall average SDI, based on all four parameters, indicated that Adélie and gentoo penguins hold the same levels of SSD, while chinstrap penguins are slightly less dimorphic. However, when excluding body mass from these calculations by considering only culmen and flipper features, the overall average SDI suggested that chinstrap penguins were most sexually dimorphic, followed by gentoo penguins, with Adélie penguins being the least dimorphic of all three species. Body mass variation is closely coupled with the seasonal energetic requirements of migration, reproduction, and molt, therefore, the overall average SDI calculated that excludes body mass is a more robust estimate of SSD that is relevant throughout the annual cycle. Within this context, although based on a different SDI, our results corroborate those by Polito et al. [66] suggesting that chinstrap penguins are the most sexually size dimorphic *Pygoscelis* species based on bill features only.

Ecological niche divergence by the sexes is thought to evolve often as a consequence of existing SSD [6], which might facilitate sexual segregation particularly when resources are limited [19], [24]. Within this context, and following our more conservative and robust overall average SDI, we would expect chinstrap penguins to show the greatest difference, and Adélie penguins the least difference, in sex-specific foraging niche. Our trophic foraging analyses, based on δ^15^N signatures of penguin RBCs follow this prediction. Male and female chinstrap penguins showed the greatest difference in pre-breeding trophic niche (∼0.2‰), male and female gentoo penguins foraged at a slightly more similar trophic niche (∼0.1‰), while there was no difference in pre-breeding trophic niche of male and female Adélie penguins. Signatures of δ^15^N were depleted in female chinstrap and gentoo penguins in comparison with their male counterparts, indicating that females foraged at a slightly lower trophic level during the 30–60 days prior to egg laying. Female chinstrap and gentoo penguins were only very slightly depleted in δ^15^N, which may reflect females relying on a slightly higher percentage of certain prey items such as Antarctic krill (*Euphausia superba*) that are relatively depleted in δ^15^N in comparison with many Antarctic fishes [68] and even other species of Southern Ocean krill (K.B. Gorman unpubl. data). In terms of δ^13^C foraging niche, there was no evidence that males and females foraged differently as the only parameter that accounted for variation in δ^13^C signatures of penguin RBCs at the one-egg stage was the year term, which accounted for a high percentage of variation in Adélie and gentoo penguins only. Studies examining isotopic differences between male and female penguins is a growing body of literature [18], [26], [50], [69]–[72]. Dehnhard et al. [26] sampled southern rockhopper penguins (*Eudyptes chrysocome chrysocome*) during a similar time period to capture pre-breeding foraging niche as in our study, in addition to work on macaroni penguins [50], [70], [72] as well as eastern (*E. filholi*) and northern (*E. moseleyi*) rockhopper penguins [71]. Studies have shown male Magellanic, gentoo, and southern rockhopper penguins to be enriched in δ^15^N relative to female conspecifics, similar to our own results. One other study examined pre-breeding foraging niche of western AP Adélie and gentoo penguins, however, the study was based on isotopic signatures of eggshells, and therefore, could not address differences between males and females of these species [73].

Our study revealed important variation in pre-breeding δ^15^N foraging niche across years, both within and among species. However, we did not detect strong support for the hypothesis that sex-specific trophic foraging interacted with yearly environmental conditions. Pre-breeding trophic foraging by male and female Adélie penguins was similarly enriched in years of relatively higher sea ice conditions, and relatively depleted in 2008, the year characterized by the lowest sea ice conditions. This result suggests that Adélie penguins were foraging at a lower trophic level, possibly including a slightly higher percentage of prey items that are relatively depleted in δ^15^N such as Antarctic krill [68], in association with lower winter average sea ice conditions. This result supports the idea that Adélie penguins inhabiting the western AP region of Antarctica are truly sea ice obligate, and from the perspective of foraging, are relying on prey items linked to the seasonal variability of winter sea ice. Conversely, in the first two years of study, gentoo penguins were relatively more enriched in δ^15^N following the 2008 winter of extremely low average winter sea ice, which suggests their pre-breeding trophic response to winter environmental variability is opposite that of Adélie penguins. However, although gentoo penguins were relatively depleted in the third year of the study characterized by the highest average sea ice conditions, individuals were not as greatly depleted as in year one, suggesting that their pre-breeding trophic foraging response was not as tightly coupled across years to the seasonal variability of winter sea ice as was detected for Adélie penguins. Even more interesting, yearly variability in chinstrap penguin pre-breeding δ^15^N foraging niche appeared entirely independent of winter sea ice conditions as males and females were increasingly enriched in δ^15^N across years. *Pygoscelis* penguins within our study population varied in their nest initiation dates both among species and years during the course of our study (see Table S1). This aspect of *Pygoscelis* penguin breeding ecology suggests that variation in pre-breeding δ^15^N foraging niche may have reflected, to some degree, the various conditions experienced during the differing time periods at which the 30–60 days prior to breeding occurred across species and years. However, the fact that each species winters and subsequently migrates to breeding rookeries from disparate areas along the western AP is likely a stronger determinant of variation in pre-breeding δ^15^N foraging niche than temporal variation in arrival and timing of nest initiation.

We have demonstrated key trophic pathways linking *Pygoscelis* penguins with environmental variability in the form of winter sea ice where Adélie penguin trophic foraging is most tightly coupled, and that of chinstrap penguins least tightly coupled, with winter environmental conditions likely reflecting the affinity each species holds during late winter and spring for sea ice habitats [29], [33]–[35], [52]. Furthermore, pre-breeding trophic foraging by Adélie and gentoo penguins appears generally divergent in response to winter environmental variability, which is interesting within the context that these two species have shown the greatest breeding population change, characterized by opposing trajectories, among our study populations of the Palmer Archipelago [36], see also [74] Figure 7. Although male and female chinstrap and gentoo penguins were sexually segregated in their pre-breeding trophic foraging niche, the absolute difference was not large enough to represent an entire trophic level, i.e., 3–4‰ for δ^15^N [21], [22], suggesting that males and females within a species should respond similarly in terms of trophic foraging to changes in future winter environmental conditions.

## Supporting Information

Figure S1 **Examples of PCR bands from both P2/P8 and 2550F/2718R primers for three mated adult pairs of** ***Pygoscelis*** **penguins.**
(PDF)

Table S1 **Average nest initiation dates of** ***Pygoscelis*** **penguins relative to November 1 for each study year.**
(PDF)

Text S1 **PCR methods.**
(PDF)

Text S2 **PCR optimization with P2/P8 primers.**
(PDF)

Text S3 **Misclassification details for probability estimates of adult** ***Pygoscelis*** **penguin sex.**
(PDF)
