# Supplementary material for: Ecological Sexual Dimorphism and Environmental Variability within a Community of Antarctic Penguins (Genus Pygoscelis)
Source: PLoS One. 2014 Mar 5;9(3):e90081. doi: 10.1371/journal.pone.0090081 (PMC3943793; doi:10.1371/journal.pone.0090081)
Supplement: Figure S1 — Examples of PCR bands from both P2/P8 and 2550F/2718R primers for three mated adult pairs of Pygoscelis penguins. (PDF) [file pone.0090081.s001.pdf]

Figure S1.

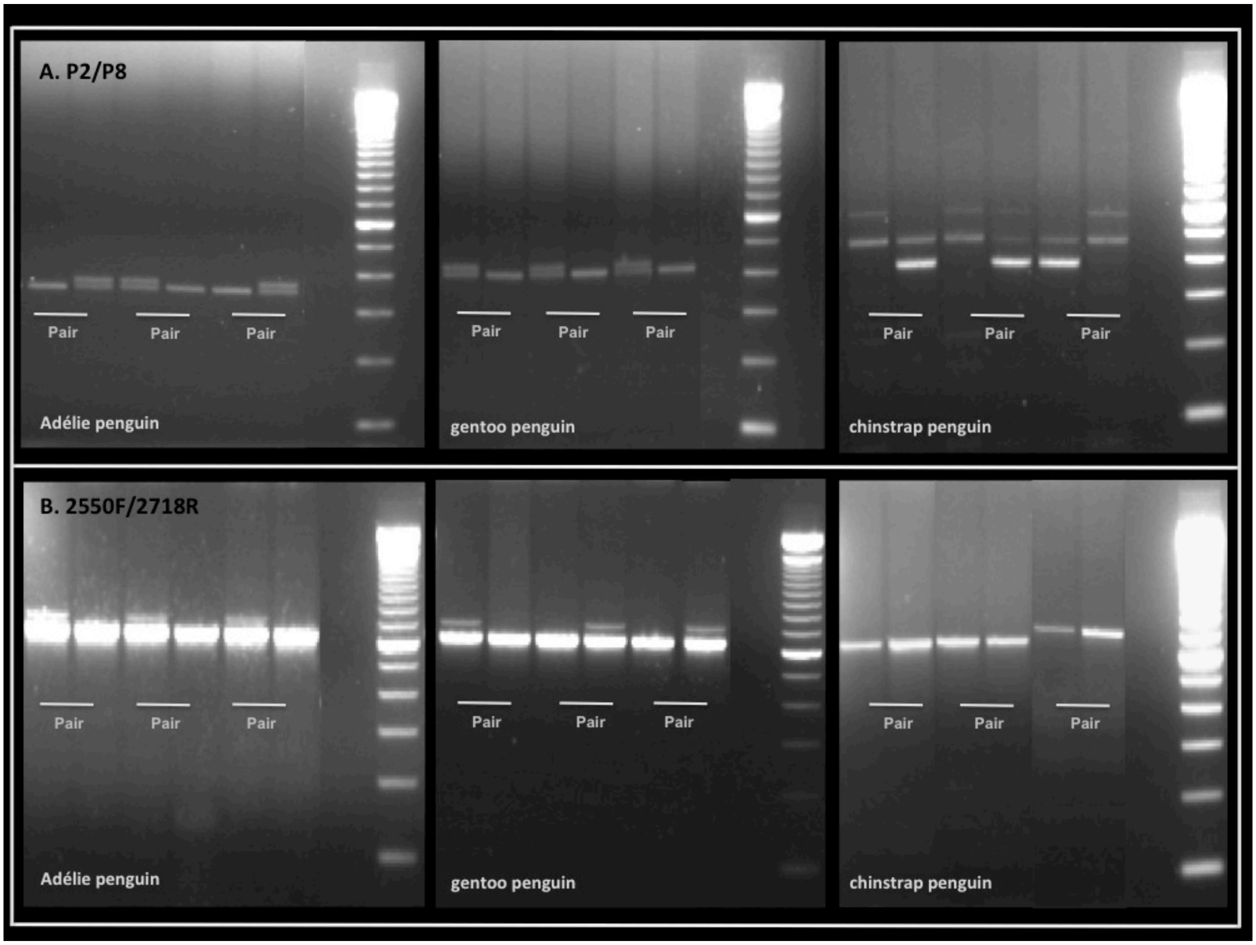

Examples of PCR bands from both P2/P8 and 2550F/2718R primers for three mated adult pairs of *Pygoscelis* penguins. P2/P8 primers correctly identified 100% of each species' pairs, while 2550F/2718R primers correctly identified 60% of Adélie and gentoo penguin pairs and 0% of chinstrap penguin pairs. The light upper P2/P8 band for chinstrap penguins was considered non-specific, as it was present in both male and female samples as shown above. Adult pairs per species shown here are not the same between primer sets.
