# Supplementary material for: Ecological Sexual Dimorphism and Environmental Variability within a Community of Antarctic Penguins (Genus Pygoscelis)
Source: PLoS One. 2014 Mar 5;9(3):e90081. doi: 10.1371/journal.pone.0090081 (PMC3943793; doi:10.1371/journal.pone.0090081)
Supplement: Table S1 — Average nest initiation dates of Pygoscelis penguins relative to November 1 for each study year. (PDF) [file pone.0090081.s002.pdf]

Table S1. Average nest initiation dates of *Pygoscelis* penguins relative to November 1 for each study year. Date paired adult penguins with a full clutch (two eggs) were observed with the first egg.

Species	Study year	Average nest initiation date	± 1SE	*n*
Adélie penguin	2007/08	13	0.45	44
	2008/09	10	0.57	48
	2009/10	16	0.46	46
chinstrap penguin	2007/08	26	0.95	18
	2008/09	20	1.41	14
	2009/10	22	0.70	22
gentoo penguin	2007/08	28	0.57	32
	2008/09	6	0.56	42
	2009/10	22	0.93	42

Abbreviations: ± 1SE = plus or minus 1 standard error, *n* = sample size.
