# Supplementary material for: Ecological Sexual Dimorphism and Environmental Variability within a Community of Antarctic Penguins (Genus Pygoscelis)
Source: PLoS One. 2014 Mar 5;9(3):e90081. doi: 10.1371/journal.pone.0090081 (PMC3943793; doi:10.1371/journal.pone.0090081)
Supplement: Text S1 — PCR methods. (PDF) [file pone.0090081.s003.pdf]

Text S1. PCR Methods. Genomic DNA was extracted from collected samples of whole blood smeared on filter paper using Insta-Gene Matrix (Bio-Rad, Hercules, CA) following standard protocols. The PCR primers P2 (5'-TCTGCATCGCTAAATCCTTT-3') and P8 (5'-CTCCCAAGGATGAGRAAYTG-3', where R=A/G, Y=T/C), as well as 2550F (5'-GTTACTGATTCGTCTACGAGA-3') and 2718R (5'-ATTGAAATGATCCAGTGCTTG-3') were both used to amplify regions of the CHD-Z and CHD-W genes. PCR was carried out using a total volume of 25 µl (15 µl PCR mix added to 10 µl genomic DNA) where PCR mix consisted of 10x PCR reaction buffer minus Mg (55 µl), 50 mM MgCl2 (44 µl), Taq polymerase (5.5 µl, 5 units/µl, invitrogen), dNTP mix (11 µl, 10 mM each, Fermentas), P2/P8 or 2550F/2718R primers (44 µl of 20 µM working stock, invitrogen), and autoclaved distilled water (156.5 µl). Using an Eppendorf Thermal Cycler (Mastercycler personal), the following thermal profile was used for P2/P8 primers as outlined by Griffiths et al. [53] including a slight modification for an initial hot start where the lid and thermoblock were pre-heated to 105 and 85 ºC, respectively, before loading samples; an initial denaturing step at 94 ºC for 1 minute (min) 30 seconds (sec) was followed by 30 cycles of 48 ºC for 45 sec, 72 ºC for 45 sec, and 94 ºC for 30 sec, followed by a final cycle of 48 ºC for 1 min and 72 ºC for 5 min. The thermal profile for 2550F/2718R followed Fridolfsson and Ellegren [54] and was modified as described above to include an hot start; the initial denaturing step at 94 ºC for 2 min was followed by a touch-down method where the annealing temperature starting at 60 ºC was lowered 1 ºC per cycle until a temperature of 50 ºC was reached. Denaturation began at 94 ºC for 30 sec, followed by annealing for 30 sec, and extension at 72ºC for 35 sec. Twenty-nine additional cycles were run after reaching the final cycle at the 50 ºC annealing temperature (i.e., 30 cycles total of 94 ºC for 30 sec, 50 ºC for 30 sec, and 72ºC for 35 sec). A final extension step included holding at 72 ºC for 5 min. After both

P2/P8 and 2550F/2718R thermal profiles were completed, samples were held at 4 ºC. Loading buffer (2.5 µl) was added to PCR products, with 10 µl then separated by electrophoresis for 120 min, at 90 V and 200 mA, using a 3% agarose gel stained with ethidium bromide, and visualized under ultra-violet light.
