# Supplementary material for: Ecological Sexual Dimorphism and Environmental Variability within a Community of Antarctic Penguins (Genus Pygoscelis)
Source: PLoS One. 2014 Mar 5;9(3):e90081. doi: 10.1371/journal.pone.0090081 (PMC3943793; doi:10.1371/journal.pone.0090081)
Supplement: Text S2 — PCR optimization with P2/P8 primers. (PDF) [file pone.0090081.s004.pdf]

Text S2. PCR optimization with P2/P8 primers. During PCR optimization with P2/P8 primers, we found that for Adélie and gentoo samples the volume of *Taq* polymerase within the PCR mix could be reduced to 1.8 *u*l without any effect to the quality of DNA amplification, but for chinstraps it was best to use the original volume of 5.5 *u*l. In addition, for chinstrap samples, the PCR thermal profile for P2/P8 primers was modified to increase the time of the initial denaturing step at 94 ºC from 1 min 30 sec to 5 min, followed by 40, instead of 30 cycles, of 48 ºC for 45 sec, 72 ºC for 45 sec, and 94 ºC for 30 sec.
