# Supplementary material for: Ecological Sexual Dimorphism and Environmental Variability within a Community of Antarctic Penguins (Genus Pygoscelis)
Source: PLoS One. 2014 Mar 5;9(3):e90081. doi: 10.1371/journal.pone.0090081 (PMC3943793; doi:10.1371/journal.pone.0090081)
Supplement: Text S3 — Misclassification details for probability estimates of adult Pygoscelis penguin sex. (PDF) [file pone.0090081.s005.pdf]

Text S3. Misclassification details for probability estimates of adult *Pygoscelis* penguin sex.

Of the five Adélie penguin individuals misclassified, four were females based on molecular data that were classified as males based on structural data for the first and third supported models. For the second best-supported model, three females and two males were incorrectly classified. The one chinstrap penguin individual misclassified was a male. The four gentoo penguin individuals misclassified were two males and two females.
